# Prognostic impact of physical activity patterns after percutaneous coronary intervention. Protocol for a prospective longitudinal cohort. The PIPAP study

**DOI:** 10.3389/fcvm.2022.976539

**Published:** 2022-09-30

**Authors:** Nathalia Gonzalez-Jaramillo, Prisca Eser, Flurina Casanova, Arjola Bano, Oscar H. Franco, Stephan Windecker, Lorenz Räber, Matthias Wilhelm

**Affiliations:** ^1^Department of Cardiology, Inselspital, Bern University Hospital, University of Bern, Bern, Switzerland; ^2^Institute of Social and Preventive Medicine, University of Bern, Bern, Switzerland; ^3^Graduate School for Health Sciences, University of Bern, Bern, Switzerland

**Keywords:** cohort, protocol, prevention, coronary disease - epidemiology, accelerometer

## Abstract

**Introduction:**

Current guidelines recommend wearable activity trackers to detect insufficient physical activity (PA) and help increase PA to prevent or ameliorate cardiovascular disease. However, there is a paucity of data regarding how objectively measured PA trajectories, patterns, and sedentary time, are associated with mortality and recurrent events after percutaneous coronary intervention (PCI) in patients with established coronary artery disease (CAD). Additionally, it remains unclear if early PA and sedentary time after PCI are associated with such outcomes. Therefore, in the present study (ClinicalTrials.gov Identifier: NCT04663373), we aim to establish the associations of objectively measured PA with major adverse cardiac events and mortality at one-year follow-up.

**Methods and analysis:**

In this single-centre observational study, patients with CAD will be prospectively recruited immediately after PCI. All the information from the clinical history, baseline characteristics, and outcomes during follow-up will be obtained from the CARDIOBASE registry. Accelerometer data will be collected for 18 days following hospital discharge and 14 days at one-year follow-up. PA trajectories will be identified by group-based trajectory modeling. Major adverse cardiac events and mortality will be prospectively monitored up to 1 year after PCI. All data will be collected using Research Electronic Data Capture.

## Introduction

Coronary artery disease (CAD) is the leading cause of cardiovascular disease (CVD) mortality (1, 2). Lifestyle physical activity (PA) and exercise-based cardiac rehabilitation (CR) successfully reduce the risk of CVD morbidity and mortality and improve the quality of life in patients with CAD (3–5). American and European guidelines (6, 7) thus recommend CR and PA for patients with acute and chronic coronary syndromes, particularly those who undergo percutaneous coronary intervention (PCI) (8).

The use of accelerometers provides objective, feasible, and reliable PA data across its spectrum, including sedentary time, activity counts (divided into time spent in light, moderate and vigorous activity), daily steps, and sleep (9). Some evidence has provided insights into the associations of objectively measured PA and short-term prognosis after hospital admission in patients with CVD. For instance, the number of steps during the three last days of hospital stay after coronary artery bypass grafting is a strong predictor for 30-day cardiac rehospitalization (10). Likewise, in patients hospitalized for decompensated heart failure, physical inactivity within the first week after hospital discharge is associated with 30-day hospital readmission (11).

Current guidelines recommend wearable activity trackers as a tool that may help increase PA (12). However, there is a paucity of data regarding how objectively measured PA patterns and sedentary time are associated with mortality and recurrent events after PCI in patients with CAD. Additionally, it remains unclear if an early increase in PA and a decrease in sedentary time after PCI is associated with such outcomes at 1-year follow-up. Therefore, in the present study (ClinicalTrials.gov Identifier: NCT04663373), we aim first to quantify PA levels, sedentary time, and PA trajectories in participants wearing an accelerometer for 18 days starting on the day of their hospital discharge and for 14 days on 1 year of follow-up. We also aim to evaluate how PA, sedentary time, and PA trajectories early after PCI are associated with major adverse cardiac and cerebrovascular events (MACCE) and 1-year mortality in patients with CAD. Through the extension of the current knowledge and understanding of the role of PA in the prognosis of CAD, these results could enhance cardiovascular health and clinical practice. Our study could emphasize the importance of objectively measured PA and sedentary time as a standard-of-care evaluation in patients with CAD to contribute to clinical decision-making, design tailored interventions and perhaps establish a prognosis.

### Objectives

Among patients with CAD who underwent PCI, we aim to establish the association of objectively measured PA and sedentary time with mortality and MACCE at 1-year follow-up.

The aim will be addressed *via* specific objectives:

(1) Determine the correlation between objectively measured PA, daily step counts, step cadence, inactivity time and sleep time over the 18 days following hospital discharge after PCI and over 14 days at 1-year follow-up.
(2) Compare PA, steps and inactivity between patients subsequently performing CR with patients not performing CR.
(3) Identify PA components and PA trajectories associated with mortality and the first occurrence of MACCE at 12 months.
(4) Identify factors that are associated with different PA trajectories from the immediate period following PCI to 1-year follow-up.

## Methods and analysis

### Study design and setting

We will conduct an observational, longitudinal monocentric study with a prospective collection of data from the University Hospital of Bern (Inselspital).

### Population

Consecutive adult patients with acute or chronic coronary syndrome who underwent PCI.

### Selection criteria

#### Inclusion criteria

(1) Men and women older than 18 years after treatment with PCI for chronic or acute coronary syndrome.
(2) Willingness to participate in the study.

#### Exclusion criteria

(1) No adequate understanding of how to wear the accelerometer.
(2) Behavioral or cognitive disorders are sufficient to interfere with the patient’s ability to comply with the protocol instructions or follow-up procedures.
(3) Frailty or extensive nursing care needs.
(4) Major surgery, including implantation of cardiac devices, or major neurologic events and cerebrovascular events, within 30 days before screening.
(5) Planned major surgery within the next 20 days after PCI, including staged cardiac surgery.

### Sample size calculation

The sample size was calculated based on the available information from the CARDIOBASE registry regarding the proportion of incident cardiovascular events among CAD patients undergoing PCI. The Schoenfeld formula was utilized for survival analysis. Based on the data from the CARDIOBASE registry, we assumed that the proportion of MACCE events within 1 year after the PCI procedure would be 9% (13). We assumed that the physically active patients in the present study would have a 50% reduction in the 1-year risk of incident MACCE, compared to the inactive ones (14). To obtain a power of 80% and a precision of 5%, we estimated a sample size of 726 participants.

### Exposures, outcomes, and covariates

#### Exposures

(a) Each component of the continuum of PA, including sedentary behavior, light, and moderate to vigorous (MVPA) levels of activity, and sleep. Further, the number of daily steps and step cadence.
(b) Trajectories of PA parameters (as stated above) in the short-term (18 days after hospital discharge) and the long-term (hospital discharge to 1-year follow-up).

#### Outcomes

(a) Main outcomes: (a) MACCE. In line with previous literature on CVD prevention (15), the definition of MACCE will include ischemic endpoints as cardiovascular death, myocardial infarction, stent thrombosis, and stroke; and revascularization endpoints as target lesion revascularization and target vessel revascularization; (b) All-cause mortality.
(b) Secondary outcomes: Individual components of MACCE.

#### Covariates

(a) Demographic factors: Sex, age, marital status, zip code.
(b) Clinical factors: Personal history of diabetes, dyslipidemia, hypertension, smoking, alcohol intake, body mass index, and CAD presentation at PCI (acute vs. stable), CR attendance.
(c) Procedural-related factors: Number of affected vessels, number of stents, presence of left main disease, and staged angioplasty.

### Protocol activities

**FIGURE 1:**
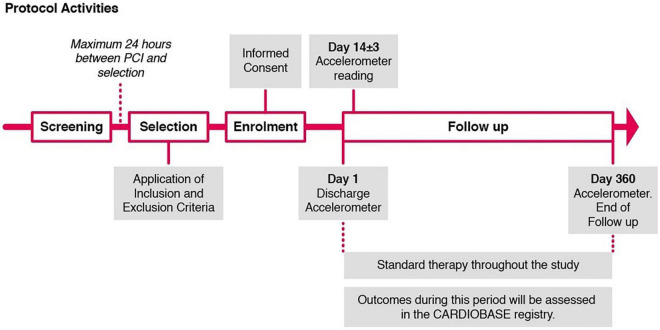
Protocol activities.

Consecutive patients who have been hospitalized for percutaneous coronary intervention (PCI) after acute or chronic coronary syndrome (ACS, CCS) will be recruited on the day of discharge or one day before discharge by a team of nurse practitioners who see the patients and inform them about available CR programs. Patients who consider participating will receive a wrist-worn accelerometer, a patient information sheet including an informed consent form and an addressed and prepaid envelope to return the signed consent form and the accelerometer after the study period. They will be asked to wear the accelerometer for 18 days starting from the day of discharge from the hospital. At discharge, the patient will start wearing the accelerometer according to the instructions given by the research team. Patients undergoing staged PCI will receive the accelerometer after the last revascularization. Patients who have their yearly routine checkup at our clinic will receive the accelerometer again after 1 year for another measuring period of 14 days (Figure 1). Patients who perform their yearly check-ups at an external cardiologist will receive the accelerometer for the follow-up measurement by mail. Detailed information concerning the occurrence of clinical outcomes (e.g., date and type of events) will be obtained from the CARDIOBASE database (NCT02241291), a prospective registry collecting all events adjudicated by an independent clinical events committee.

At each visit, all the assessments will be recorded on source documentation. A summary of the protocol activities is provided in Figure 1.

### Measures

#### Physical activity monitoring

Tri-axial accelerometers (Axivity AX-3, Axivity Ltd., Newcastle, UK) will be worn continuously on the non-dominant wrist for 18 days. The devices will be programmed with the open-source software AX3 GUI V43 (16) to record tri-axial accelerations of ±8 g at 50 Hz for 18 days starting on the evening of the day of hospital discharge. This period was chosen to achieve at least 14 days of physical activity data also from patients who are transferred to another hospital or home care before returning home. When patients return to the clinic for their follow-up visit after 1 year, they will receive again an accelerometer to be worn for 14 days. Patients completing their yearly follow-up visit with their resident cardiologist will be contacted by mail and if willing to wear the accelerometer again, they will receive it by mail with a prepaid returning envelope.

#### Data processing

Raw data will be downloaded as cwa files by AX3 GUI V43 and processed by a research-driven open-source R package named GGIR (version 2.4.0) (17, 18). Criteria for data inclusion for analysis is a minimum of 7 days of at least 12 h of daily wear time. The default acceleration metric of the package, which is the Euclidean norm (vector magnitude) minus one (ENMO), will be used for the calculations of the movement component of the raw acceleration data. It describes the conversion of the raw tri-axial acceleration data into an omnidirectional measure of body acceleration (19). The resulting ENMO values are expressed in gravity-based acceleration units (milligravity units [mg]) averaged over 5 s epochs [window sizes = c (5, 900, 3600)].

#### Physical activity levels

The following activity domains were pre-defined: (20) < 25 mg for inactivity, 25–99 mg for light physical activity [threshold.lig = c (25)] and ≥100 mg for moderate to vigorous physical activity (MVPA) [threshold.mod = c (100)]. Sleep will be identified by the longest daily time window with minimal acceleration and the least positional changes of the wrist (21, 22). Time spent at different PA levels will be accrued in 1 min bouts. During analysis, auto-calibration using local gravity as the reference will be conducted and non-wear time will be determined over a window size of 60 min with a 15-min sliding window [window sizes = c(5, 900, 3600)] (23, 24).

#### Steps

Whereas activity parameters will be derived directly from GGIR, steps will be determined by a Windowed Peak Detection open-source algorithm (Verisense_step_algorithm, last updated: 14.04.2021) based on the design of Gu et al. (25) and implemented for use in combination with the GGIR R package available on GitHub (26). The input parameters used for the step algorithm were validated in a study with 22 CVD patients during an outdoor exercise session of our CR program (27).

#### Cadence

Cadences for each minute will be calculated from the meta-data derived by the step algorithm, which includes the number of steps for each 5 s epoch. From this information, we will calculate the mean cadence over the whole 24 h cycle. Moreover, daily minutes with ≥100 steps/min and with 0 steps/min will be extracted (28). We will determine mean cadences for the most active 1, 30, and 60 min, as proposed by Tudor-Locke et al. (29).

### Possible sources of bias

Patients cannot see activity or steps on the device, as there is no display on Axivity AX3. However, inherent to the observational design of the study, a selection bias may arise if patients with low PA levels refuse to wear the accelerometer. Information bias would be expected if patients do not wear the accelerometer on days when they move less, as they are told that they will get a personal data summary after the end of their wearing time. On the other hand, patients may make an extra effort to move more due to knowing that their data will be recorded and analyzed (Hawthorne effect).

### Data analysis

#### Statistical analysis plan

All the statistical analyses will be performed in STATA 17IC and R. For all comparisons, a two-sided *p*-value < 0.05 will be considered statistically significant.

Descriptive statistics will be presented by relative frequencies for qualitative variables or by the mean and standard deviation (SD) or median and interquartile range (IQR), according to the normal distribution of quantitative variables. Unpaired *T*-tests, Chi-square tests and Mann–Whitney U tests will be used to present statistical differences in baseline characteristics according to the fulfilment of PA recommendations and the identified trajectories. If allowed by the data, we will perform logistic regression models to identify factors associated with short and long-term PA trajectories.

We will use group-based trajectory modeling (30) (GBTM) to identify distinctive clusters of individual PA trajectories and for profiling the characteristics of individuals within the trajectories. GBTM permits the analysis of the effect of time-stable covariates on the probability of group membership and the effect of time-dependent covariates on the trajectory itself (30).

We will test whether mean PA parameters of the whole observation period or trajectories could predict MACCE in addition to traditional risk factors. Furthermore, we will perform receiver operating characteristic (ROC) curves to identify a threshold of the most convenient time to start PA after coronary revascularization. Predictive values of daily steps during the first 2 weeks after hospital discharge will be quantified and tested by the increase in the area under the curve (AUC) of the ROC curve of a model consisting of traditional risk factors (age, sex, cardiovascular risk factors, cardiovascular history, and comorbidities) and time of starting PA after PCI.

We will also use Kaplan–Meier estimates to analyze the association of PA components including sedentary behavior, light, moderate, and high levels of activity, sleep, and the number of steps or step cadence with recurrent cardiovascular events after PCI. After inspection of Schoenfeld residuals to check the proportional hazards assumption, we will perform Cox regression models to compare rates of MACCE among trajectories. Furthermore, the models will be adjusted for age, sex, and clinically relevant variables, including acute vs. chronic CAD, staged PCI, and CR attendance, among others. Time-to-event will be censored at last patient contact for patients without MACCE. Multiple imputations will be performed in case of missing data.

## Data availability statement

The raw data supporting the conclusions of this article will be made available by the authors, without undue reservation.

## Ethics statement

For all research activities, informed consent will be obtained from participants. Each participant will receive information about the objectives and the procedures of the study. After study completion, all the patients will receive their summary data. Only research team members will have access to data during the study, which will be stored in REDCap, and hosted on secure servers at Inselspital. All data will be anonymized, handled and stored in line with the national data protection laws in Switzerland (31). To protect the identity of participants, they will each be assigned a participant identification number (PIN). The list linking participants’ names to their PIN will be stored separately and securely. Only the principal investigator will have access to that file. The project is approved by the cantonal ethics committee of Bern and is registered in ClinicalTrials (NCT04663373). The CARDIOBASE registry also complies with the Declaration of Helsinki and is approved by the ethics committee.

## Author contributions

NG-J, AB, OF, PE, and MW designed the study. FC involved in the patient recruitment. SW, LR, and PE were involved in the data collection. NG-J and PE drafted the manuscript. All authors approved the final version of the manuscript.
