# Erratum: Prognostic impact of physical activity patterns after percutaneous coronary intervention. Protocol for a prospective longitudinal cohort. The PIPAP study

**DOI:** 10.3389/fcvm.2023.1176600

**Published:** 2023-03-21

**Authors:** Frontiers Production Office

**Affiliations:** Frontiers Media SA, Lausanne, Switzerland

**Keywords:** cohort, protocol, prevention, coronary disease - epidemiology, accelerometer

An Erratum on Prognostic impact of physical activity patterns after percutaneous coronary intervention. Protocol for a prospective longitudinal cohort. The PIPAP study By Gonzalez-Jaramillo N, Eser P, Casanova F, Bano A, Franco OH, Windecker S, Räber L and Wilhelm M. (2022) Front. Cardiovasc. Med. 9:976539. doi: 10.3389/fcvm.2022.976539

An omission to the funding section of the original article was made in error. The following sentence has been added: “Open access funding was provided by the University Of Bern”.

The original version of this article has been updated.

